# Socioeconomic Determinants of Health-Related Quality of Life of Entrepreneurs. A Cross-Sectional Study

**DOI:** 10.3390/ijerph182212103

**Published:** 2021-11-18

**Authors:** Daniel Puciato, Michał Rozpara, Marek Bugdol, Tadeusz Borys, Teresa Słaby

**Affiliations:** 1 Faculty of Finance and Management, WSB University of Wroclaw, ul. Fabryczna 29-31, 53-609 Wrocław, Poland; 2 Institute of Sport Sciences, Akademia Wychowania Fizycznego im. Jerzego Kukuczki w Katowicach, Mikołowska 72A, 40-065 Katowice, Poland; m.rozpara@awf.katowice.pl; 3 Faculty of Management and Social Communication, Jagiellonian University, ul. Prof. Stanisława Łojasiewicza 4, 30-348 Kraków, Poland; marek.bugdol@uj.edu.pl; 4 Institute of Management and Quality, University of Zielona Góra, ul. Podgórna 50, 65-246 Zielona Góra, Poland; t.borys@wez.uz.zgora.pl; 5 Institute of Management and Technical Sciences, Warsaw Management University, 03-772 Warsaw, Poland; teresa.slaby@wsm.warszawa.pl

**Keywords:** health-related quality of life, quality of life domains, entrepreneurs, Poland

## Abstract

Background: Quality of life is one of the most important conceptual categories in many scientific fields and socio-economic practices. In the authors’ opinion, the assessment of the overall quality of life and the health-related quality of life of private entrepreneurs deserves particular attention. Until now, quality of life and its determinants in entrepreneurs have been investigated by few authors. The aim of this study was to identify and assess the key determinants of quality of life and its health-related aspects in entrepreneurs from Wroclaw, Poland. Methods: A questionnaire survey was carried out in a group of 616 entrepreneurs selected from among 4332 individuals (2276 women, 2056 men) who had participated in a study on the socioeconomic determinants of quality of life and physical activity of Wrocław residents of working age. The main research method was a diagnostic survey using S-ES and WHOQOL BREF questionnaires. Information was obtained on respondents’ quality of life and perceived health condition, as well as sex, age, education, marital status, number of people in the household, income per capita, savings, and indebtedness. The collected data were subjected to statistical analysis using numerical distribution, medians, and quartile deviation. Crude and adjusted odds ratios were used to assess relationships between entrepreneurs’ quality of life and socioeconomic status. Results: Over 66% of Wroclaw entrepreneurs rated their overall quality of life as average or above average and about 34% as below average. An average or above assessment of perceived health condition was provided by 71% of respondents, and below average by 29% of respondents. Health-related quality of life was assessed as average or above average in the environmental domain by 79%, physical domain by 77%, social domain by 65%, and psychological domain by 58% of the entrepreneurs. Among the respondents significant correlations were found between quality of life and perceived health condition; family status, i.e., marital status; number of persons in the household; and financial status, i.e., per capita income, savings, and debt. Conclusions: The results of this study can be used for managing the quality of life of entrepreneurs. Quality of life determinants should be constantly updated, as they may change along with further economic development and Poland’s economic convergence with better developed EU countries.

## 1. Introduction

**Figure 1:**
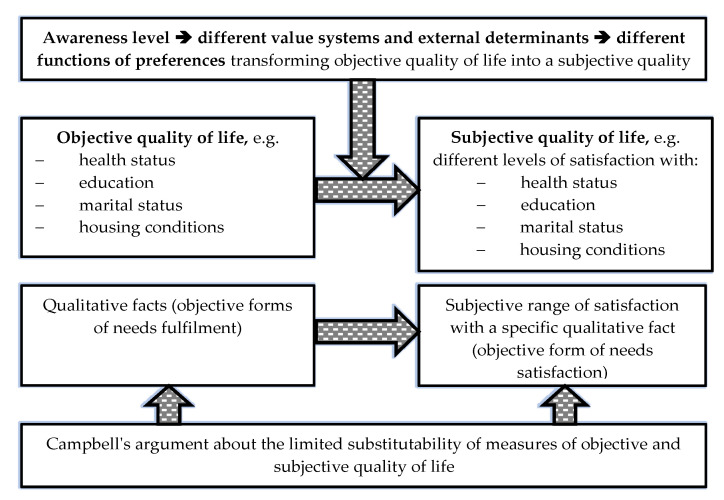
Flowchart of the transformation of objective quality of life into its subjective perceptions. Source: author’s own.

Quality of life is one of the main conceptual categories in many scientific disciplines and in socioeconomic practice. It is often used as a measure of the level of social development in comparative studies of countries or social groups [1,2,3]. According to the authors of this study, the assessment of health-related quality of life (H-RQoL) is particularly noteworthy in relation to entrepreneurs (subjective health-related quality of life is referred to in this paper as health-related quality of life, satisfaction with one’s own health, or perceived health condition). Research results indicate the very fact of starting one’s own business is significant for quality of life [4,5] in its objective and subjective dimensions (Figure 1).

However, the results of numerous studies indicate that quality of work life (QWL) is also important for entrepreneurs’ quality of life. According to Rose et al. [6], Sirgy et al. [7], Stucki and Bickenbach [8], and Subba and Neelima [9], the six most important QWL determinants include pay and stability of employment, occupational stress, work schedule, interpersonal relations, working conditions, and occupational safety. These factors affect all employees of a company in different ways, as well as entrepreneurs themselves in a specific way.

It is worth noting that the financial status of entrepreneurs, especially owners of long-established businesses, is often higher than that of non-entrepreneurs [4]. Litvin and Petrascu [10] also showed that for entrepreneurs, the implementation of investment policies is important for their quality of life. A particularly favorable impact on the quality of life occurs when the investment activity is of an innovative nature [11]. Hamilton [12] also observes that self-employment can increase the level of perceived life satisfaction. However, Charles-Leija et al. [13] observed a lower level of life satisfaction and perceived health condition in entrepreneurs, compared to their employees. Entrepreneurs are often heavily burdened with work and exposed to the negative effects of stress [14] and occupational burnout [15]. They also sometimes find it difficult to balance work and family responsibilities, which can be a cause of work–family conflict [16]. Especially since entrepreneurs not only value independence and higher income, but also leisure time and work–life balance, which can be a potential ground for internal conflict and have a negative impact on some quality of life domains [17,18].

Koponen [19] and Litwin and Phan [20] also found that political factors, including the ability to co-create social and economic assets, are also significant for the quality of life of entrepreneurs, as well as for motives for starting a business. The necessity of starting one’s own business may sometimes result from negative motives, e.g., high unemployment rates and lack of employment opportunities; job loss; low wages, which do not allow satisfying one’s needs; deterioration of the family situation; or requirements imposed by some companies, mainly corporations, such as the transfer of employees into so-called self-employment schemes. Sometimes positive motives, i.e., striving for independence, self-fulfillment, improvement of the material situation, or willingness to create new assets, are more likely to lead to starting one’s own business. In the case of the dominance of the former group of motives—a negative or neutral impact—and in the case of dominance of the latter—a neutral or positive impact on quality of life assessment can be assumed [21].

An important, but hitherto marginally considered in empirical studies, determinant of the quality of life of entrepreneurs is their socioeconomic status. Contrary to popular belief, entrepreneurs are a diverse group in this regard. For example, there may be large differences in income and wealth between the owner of a large trading company and a self-employed barber. However, previous studies have only investigated age, sex, and education level as potential predictors of entrepreneurs’ quality of life. Fatoki [22], Gherardi [23], and Rehman and Roomi [24] showed that male sex may be a significant predictor of higher quality of life for entrepreneurs. Pounder [25] observed that younger age and higher education level are associated with higher quality of life. Similar observations regarding age were made by El Shoubaki and Stephan [26], but they did not confirm any significant correlations between quality of life and education level.

Thus, the results of previous research on the socioeconomic determinants of entrepreneurs’ quality of life are not uniform. Moreover, family and material factors as potential quality of life modifiers have not been taken into account until now. It should be noted that the determinants of overall quality of life and health-related quality of life of entrepreneurs have rarely been examined. The authors of this paper believe that the assessment of health-related quality of life (H-RQoL) in this professional group deserves special attention. Thus, the choice of this research subject is justified both by its poor recognition in the literature, as well as by the more general premise related to the recognition of health-related quality of life as a key predictor of overall quality of life [8]. Reducing the identified research gaps is therefore a key objective.

The aim of this study was to examine the assessments of overall quality of life and health-related quality of life, and the relationship of these assessments to selected indices of the socioeconomic status of entrepreneurs, using the example of selected representatives of this professional group from Wrocław (Poland). Two research questions were formulated:

1. How do entrepreneurs from Wrocław rate their overall quality of life and health-related quality of life?
2. Does socioeconomic status differentiate the quality of life of Wroclaw entrepreneurs?

## 2. Materials and Methods

**Figure 2:**
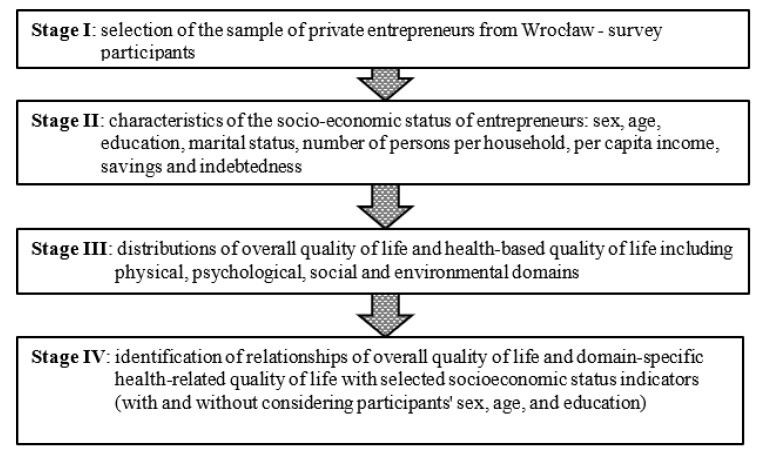
Four stages of the study. Source: author’s own.

A flowchart of the study stages is shown in Figure 2.

### 2.1. Participants

This paper is based on the results of a survey carried out in a group of 616 entrepreneurs, selected from among 4332 individuals (2276 women and 2056 men) who participated in research on the socioeconomic determinants of the quality of life and physical activity of Wrocław residents of working age. The research was carried out between 2014 and 2016.

The sample size was estimated based on the following formula [27]:

$$n=\frac{N}{1+\frac{e^{2}\left(N-1\right)}{u_{\alpha }^{2}pq}}$$

where *N*—number of Wrocław residents as of 31 December 2013 (*N* = 632,067); *p*—the fraction of Wrocław population of working age as of 31 December 2013 (*p* = 0.63); *q*—constant equal to 1—*p* (*q* = 0.37); *e*—assumed error of *p* fraction estimation (*e* = 1.5); and *u_α_*—value of standardized normal distribution *N*(0, 1) for the confidence coefficient 1 − *α* (*z_α_* = 1.96 for *α* = 0.05).

The minimum sample size was estimated at 3955 individuals, thus the number of collected questionnaires (*N* = 4332) was higher than required.

The selection of the research sample was of a multistage and mixed character (random and nonprobability sampling). In the first step, using a random number table, 10 housing estates in Wrocław were drawn. In the second step, using the same random mechanism, 3 streets were selected from each of the 10 housing estates. In the last step, from among passers-by encountered in the selected streets, every fourth person was asked to participate in the survey. The following inclusion criteria were assumed in the study: entrepreneurial status, address of residence in one of the selected streets, and working age (18–64 years). In the study a broad definition of an entrepreneur was used, which, according to Polish law, is a person conducting a business activity, including the so-called self-employed. The exclusion criteria involved pregnancy and chronic diseases, e.g., cancer, diabetes, arterial hypertension, osteoarthritis, or osteoporosis. All respondents were informed about the purpose and course of the survey and their voluntary participation. They were asked to provide informed consent to participate.

The distributions of respondents’ socioeconomic status characteristics are shown in Table 1. In the study group, men constituted 65% and women 35% of all respondents. Among the respondents, 64% were below the age of 44 years, and 36% were above 44 years. Vocational or primary education was reported by 19% of the entrepreneurs, secondary education by 31%, and higher education by 50%. A total of 17% of entrepreneurs were single and 83% were in relationships. More than 34% of the respondents lived in one- or two-person households, 58% in three- or four-person households, and more than 8% in five- or more-person households. Among the Wrocław entrepreneurs, individuals with incomes of up to USD 260 per capita accounted for 14%, and those with incomes of USD 260 or more for 86% of the respondents. Finally, 73.5% of respondents had savings, and 50.5% had debt (Table 1).

### 2.2. Methods

#### 2.2.1. Socioeconomic Background

In the course of the face-to-face interviews using the author’s S-ESQ questionnaire, data were obtained on respondents’ marital status (unmarried, married), number of persons in the household (up to 2, 3–4, 5 and above), per capita income (below USD 260, USD 260 and more), having savings (YES, NO), and debt (YES, NO), which were considered the independent variables (IVs); as well as sex (female, male), age (up to 44, 44 and above), and education (higher, secondary, primary and basic vocational) as confounding variables (CVs).

#### 2.2.2. WHOQOL

WHOQOL BREF was used to assess respondents’ overall quality of life and perceived health condition [28]. The questionnaire consisted of 26 closed questions, with answers on a five-level Likert scale. Answers to particular questionnaire items were used in accordance with the accepted data processing key to determine the following indicators: overall quality of life (1–5 pts.); perceived health condition (1–5 pts.); and health-related quality of life in four domains: physical (4–20 pts.), psychological (4–20 pts.), social (4–20 pts.), and environmental (4–20 pts.). For quality of life indicators in the physical, psychological, social, and environmental domains, the raw scores were transformed into a 4–20 point scale. Due to the fact that the distributions of the quality of life indicators differed from normal, they were expressed on a dichotomous scale. The median values of these indicators were used as the points of division in the applied categorization. A score equal to or greater than the median indicated at least an average, while a score below the median indicated a lower than average, level of values of those indicators among the surveyed entrepreneurs. The dichotomous measures of general quality of life, health-related quality of life in the physical, psychological, social, and environmental domains, and perceived health condition were treated as dependent variables (DVs).

The research project was approved by the Commission of Bioethics of the University School of Physical Education in Wrocław. The study had a cross-sectional survey design. The method of a diagnostic survey with a questionnaire technique was applied.

#### 2.2.3. Statistical Analysis

The obtained data were subjected to statistical analysis, which resulted in determining the number (*n*) and percentage (%) in the distributions of respondents within the categories of dependent and independent variables. Medians (Me) and quartile deviation (QD) for DVs were also calculated. The goodness-of-fit Chi-square test (χ2) was used to test the null hypothesis (H0) that the distribution of individual variables is uniform versus the alternative hypothesis (H1) that it is not continuous. Crude odds ratio (OR) and the Mantel–Haenszel (adjusted) odds ratio (aOR) were used to assess relationships between overall quality of life; perceived health condition; quality of life in its physical, psychological, social, and environmental domains; and socioeconomic status characteristics. The predictive value of the analyzed models was assessed with the area under the ROC curve (AUC). The level of statistical significance was set at α = 0.05. All statistical calculations were made using the IBM SPSS Statistics 26 software package (IBM Corporation, Armonk, NY, USA).

## 3. Results

### 3.1. Quality of Life and Perceived Health Condition Assessment

In the light of the dichotomous classification of quality of life and perceived health measures, 66% of the Wroclaw entrepreneurs characterized their overall quality of life as average or above average and 34% as below average. An average or above average level of perceived health condition was reported by 71% of respondents and below average by 29% of respondents. A total of 79% of the entrepreneurs reported average or above average levels of health-related quality of life in the environmental domain, 77% in the physical domain, 65% in the social domain, and 58% in the psychological domain. The above differences were statistically significant (*p* < 0.001) (Table 2).

### 3.2. Overall Quality of Life in Terms of Socioeconomic Status

Table 3 presents the results of analyses of the relationships between overall quality of life and selected factors of socioeconomic status among the surveyed entrepreneurs, without and with consideration of their gender, age, and education. The adjusted odds ratios of at least an average level of overall quality of life were slightly more than twice as high (aOR = 2.84, 1.44–5.59) in entrepreneurs living in households with up to two people, and more than three times as high (aOR = 3.47, 1.82–6.62) in those living in 3–4 person households, than in respondents from households with five or more people. The odds of average or above overall quality of life were more than three-and-a-half times higher in respondents with incomes at or above USD 260 per person than in respondents with incomes below USD 260 (aOR = 3.74, CI: 1.85–7.56). Entrepreneurs with financial savings were 80% more likely than those without savings to report at least an average level of overall quality of life. The lower limit of the confidence interval for aOR for this variable was 1.94, while the upper limit was 2.71 (Table 3).

### 3.3. Perceived Health Condition in Terms of Socioeconomic Status

Socioeconomic status determinants of the perceived health condition of entrepreneurs in Wroclaw were, after adjusting for sex, age, and education, per capita income and having savings. The odds of having an average or higher level of perceived health condition were more than 20 times higher (aOR = 23.84) in respondents with per capita incomes at or above USD 260 compared with respondents with incomes below USD 260, and more than two and a half times higher (aOR = 2.57) in those with savings compared with those without savings (Table 4).

### 3.4. Health-Related Quality of Life in the Physical, Psychological, Social, and Environmental Domains in Terms of Socioeconomic Status

Table 5 and Table 6 present the results of the analysis of relationships between health-related quality of life in its physical, psychological, social, and environmental domains and selected variables of the socioeconomic status of Wroclaw entrepreneurs, with and without considering their gender, age, and education.

Marital status, number of persons in the household, income per capita, savings, and debt were independent variables significantly determining respondents’ perception of their own health in the physical domain. The adjusted odds ratio of at least an average level of quality of life in this domain was 45% lower (aOR = 0.55) in entrepreneurs living alone than in couples, more than four and a half times higher (aOR = 4.51) in those living in 1–2 person households, and seven times higher (aOR = 7.08) in 3–4 person households than in respondents living in 5- or more person households. The odds of at least an average rating of quality of life in the physical domain were slightly more than twice as high (aOR = 2.29) in those with savings than in those without savings, and two-thirds lower (aOR = 0.34) in those with debt than in those without debt (Table 5).

The respondents’ health-related quality of life in the psychological domain was significantly associated with the number of persons in the household. The adjusted odds of average or above average quality of life in this domain for those living in 1–2 person households was more than thirty-two times higher (aOR = 32.44), and for those in 3–4 person households it was twenty-one and a half times higher (aOR = 21.51), than for respondents living in five- or more person households (Table 5).

The number of people in the household, per capita income, and savings were significant determinants of the health-related quality of life of entrepreneurs in the social domain. The odds of an average or above assessment of quality of life in this domain were 56% lower for entrepreneurs living in 1–2 person households (aOR = 0.44) and 54% lower for entrepreneurs living in 3–4 person (aOR = 0.46) compared to those living in five or more person households. The adjusted odds of at least average quality of life in the social domain were more than two and a half times higher (aOR = 2.77) in entrepreneurs with per capita incomes equal to or higher than USD 260 than in respondents with incomes below USD 260. Entrepreneurs with savings were more than four and a half times more likely (aOR = 4.81) than those without savings to report at least an average rating of health-related quality of life in the social domain (Table 6).

Among the Wrocław entrepreneurs, health-related quality of life in the environmental domain was significantly correlated with per capita income and savings. The adjusted odds of average or above average health-related quality of life in the environmental domain were nearly seventeen times higher (aOR = 16.83) in respondents with incomes equal to or above USD 260 than in respondents with lower incomes. Entrepreneurs with savings were just over six times more likely (aOR = 6.27) than those without savings to report at least average quality of life in the environmental domain (Table 6).

## 4. Discussion

The study results show that the majority of the surveyed entrepreneurs assessed their overall quality of life, perceived health condition, and health-related quality of life in the physical, psychological, social, and environmental domains as at least average. Some previous studies also indicated a relatively high quality of life for entrepreneurs compared to representatives of other professional groups [4,5,10,11]. Financial advantages [4], possibilities of development [11], and independence [13] are indicated as the main reasons for the high quality of life of entrepreneurs. However, it should be mentioned that Charles-Leija et al. [13] reported lower quality of life scores for entrepreneurs compared to their employees. The analysis of literature indicates that this may be due to the heavy workload and stress [14] and the difficulty of reconciling professional responsibilities with other areas of life, mainly family life and leisure time [16,17,18,29]. Therefore, the problem of assessing the quality of life of entrepreneurs has not yet been completely resolved.

The assessment of overall quality of life, perceived health condition, and health-related quality of life in the physical, psychological, social, and environmental domains by entrepreneurs from Wrocław was differentiated by marital status, number of people in the household, per capita income, savings, and debt.

People in relationships, compared to those living alone, were more likely to report at least an average level of health-related quality of life in the physical domain. Glenn and Weaver [30] indicate that marriage contributes to well-being for both men and women. Statistically significant associations between quality of life and marital status were also noted by Povey, Boreham, and Tomaszewski [31]. Voss, Floderus, and Diderichsen [32] also showed that divorce often contributes to problems in the workplace and, consequently, to lower quality of life scores. At the time of the study every third married couple in Poland had divorced [33], so some of the unmarried respondents were likely to have experienced divorce.

As the number of people in the household increases, the odds of reporting at least an average overall quality of life and health-related quality of life in the physical and psychological domains decreased, but this increased in the social domain. The results of some studies indicate that having children may sometimes reduce the quality of life of both sexes [30]. In modern societies working parents often face difficulties in trying to balance work and family responsibilities. This can have a negative impact on quality of life and cause stress, especially when having a large family. This is a likely explanation for the decrease in quality of life scores with the increasing number of people in the household, as found in our study. Moreover, a large number of people in the household may also include elderly or sick people in the household. As shown by some authors, the numerous problems associated with caring for the elderly and the sick may cause health problems and deterioration in the quality of life of caregivers, especially in the physical and psychological domains [34,35,36].

A higher per capita income in an entrepreneurs’ household was associated with a higher likelihood of at least average overall quality of life, perceived health condition, and quality of life in the social and environmental domains. Positive associations of overall quality of life with income levels have already been noted in empirical studies. Kulik et al. [37] showed that income, in addition to variables such as place of residence and professional activities, has a significant impact on the quality of life of people of working age. Yasartürk, Akyüz, and Gönülates [38] reported positive correlations between leisure satisfaction, quality of life, and family and personal income. Monthly income and health behaviors are also primary predictors of quality of life, as observed by Kooi-Yau Chean et al. [39]. The income level and employment status were significant predictors of quality of life in Povey, Boreham, and Tomaszewski [31]. The significant impact of economic security, including income and medical insurance coverage, on health-related quality of life was also confirmed by Chiu and Yang [40]. Some studies indicated that particularly strong positive associations of health-related quality of life with income can be found in countries with a medium level of economic development, including Poland. For example, Frijters, Haisken-DeNew, and Shields [41] noted that higher real household income led to a significant increase in the quality of life of the inhabitants of East Germany following reunification. Research findings also indicate that economic deterioration and associated stress are almost always linked with a decrease in quality of life. This is particularly evident in situations of economic crisis and may even be associated with an increase in human mortality [42,43].

The conditional probability of at least average levels of overall quality of life, perceived health condition, and health-related quality of life in the physical, social, and environmental domains was higher in entrepreneurs with savings compared to those without savings. Yodmai, Somrongthong, and Kumar [44], in their study of the quality of life of the elderly in Thailand, concluded that sufficient income, savings, and healthcare services are significant quality of life determinants. According to Clarke et al. [45], the amount of savings, e.g., in retirement plans, is also often related to the expected quality of life.

Entrepreneurs with debt were significantly less likely to report at least average health-related quality of life levels in the physical domain than those with no debt. Results of previous studies of different social groups indicated that debt had a large and negative impact on quality of life, and which is higher in women than in men [46,47]. However, earlier studies did not apply to entrepreneurs, for whom financing business activities from external sources may, on the one hand, testify to the dynamic development of the company, on the other hand, be associated with certain liabilities to creditors. Debt (credits and loans) can therefore affect the quality of life of entrepreneurs both negatively and positively. The positive impact of loans on students’ quality of life was reported by Mansilla Chiguay, Denegri Coria and Álvarez Escobar [46], Chisholm-Burns et al. [47], and Daniels [48]. Moreover, debt is usually associated with an increase in current consumption, at the expense of future consumption, which, at least in the short term, can have a positive impact on quality of life, especially in its psychological domain. The results of the present study seem to confirm this tendency. Among the entrepreneurs from Wroclaw, at least an average level of quality of life in the psychological domain was more often reported by those with debt than those without it. According to Cook and Garrett [49], the direction and strength of the correlation of quality of life assessment with debt also depends on factors such as expectations towards life, experiences, or tolerance of uncertainty. The last element, in particular, is one of the most important attributes of a successful entrepreneur.

### Strengths and Limitations

This study has its strengths and weaknesses. Its strength is the selected study group; since, the quality of life of entrepreneurs, especially in post-communist countries, has rarely been analyzed. Moreover, potential modifiers of entrepreneurs’ quality of life such as marital status, number of people in the household, income, savings, or debt, with and without considering the respondents’ gender, age, and education, had not been investigated previously. A weakness of the study is the limitation of its spatial scope to the residents of only one city. Future research should involve a research population representative of Poland as a whole and even include populations from other Central and Eastern European countries. Cross-sectional research should also be replaced by continuous research.

## 5. Conclusions

The survey findings showed that most of the entrepreneurs participating in the study rated their quality of life as average or above average. Among the respondents, significant relationships were noted between quality of life and family situation: marital status and number of people in the household; and material situation: income per capita, savings, and debt. Improving the quality of life of representatives of different social groups should be one of the strategic objectives of state socioeconomic policy. This seems to be particularly relevant for entrepreneurs, who generate most of the gross domestic product (GDP); and their economic activity is of considerable importance for the labor market and, consequently, for the quality of life of the rest of society. It is also necessary to constantly monitor the quality of life of entrepreneurs and its socioeconomic determinants. The results of previous studies indicate that these modifiers may change, especially in a situation of further dynamic economic development and Poland’s attainment of economic convergence with better developed countries.

## Figures and Tables

**Table 1:** Socioeconomic characteristics of private entrepreneurs from Wrocław (*N* = 616).

Variable		*n* (%)
Sex	Female	216 (35.1) ***
	Male	400 64.9)
Age	Below 44 years	396 (64.3) ***
	Above 44 years	220 (35.7)
Education	Higher	310 (50.3) ***
	Secondary	192 (31.2)
	Primary	114 (18.5)
Marital status	Unmarried	107 (17.4) ***
	Married	509 (82.6)
Persons per household	≤2 persons	210 (34.1) ***
	3–4 persons	354 (57.5)
	≥5 persons	52 (8.4)
Per capita income	≥260 USD	529 (85.9) ***
	<260 USD	87 (14.1)
Savings	Yes	453 (73.5) ***
	No	163 (26.5)
Indebtedness	Yes	311 (50.5)
	No	305 (49.5)

Notes: *n*—number of participants; %—percent of participants. *** *p* ≤ 0.001.

**Table 2:** Quality of life and perceived health condition in private entrepreneurs from Wrocław (*N* = 616).

Variable		*n* (%)
Overall Quality of Life	Average and above	408 (66.2) ***
	Below average	208 (33.8)
Perceived Health Condition	Average and above	438 (71.1) ***
	Below average	178 (28.9)
Physical Domain	Average and above	477 (77.4) ***
	Below average	139 (22.6)
Psychological Domain	Average and above	357 (58.0) ***
	Below average	259 (42.0)
Social Domain	Average and above	400 (64.9) ***
	Below average	216 (35.1)
Environmental Domain	Average and above	488 (79.2) ***
	Below average	128 (20.8)

Notes: *n*—number of respondents; %—percent of respondents. *** *p* ≤ 0.001.

**Table 3:** Overall quality of life and socioeconomic status of private entrepreneurs from Wrocław (*N* = 616).

Variable		Overall Quality of Life		OR (±95% CI)	AUC	aOR (±95% CI)	AUC
		Average and Above *n* (%)	Below Average *n* (%)				
Marital status	Unmarried	74 (69.2)	33 (30.8)	1.17 (0.75–1.84)	0.51	1.19 (0.74–1.92)	0.55
	Married	334 (65.6)	175 (34.4)	1.00		1.00	
Persons per household	≤2 persons	131 (62.4)	79 (37.6)	1.93 * (1.05–3.57)	0.55	2.84 ** (1.44–5.59)	0.74
	3–4 persons	253 (71.5)	101 (28.5)	2.92 *** (1.62–5.28)	0.57	3.47 *** (1.82–6.62)	071
	≥5 persons	24 (46.2)	28 (53.8)	1.00		1.00	
Per capita income	≥260 USD	372 (70.3)	157 (29.7)	3.36 *** (2.11–5.35)	0.58	3.74 *** (1.85–7.56)	0.65
	<260 USD	36 (41.4)	51 (58.6)	1.00		1.00	
Savings	Yes	315 (69.5)	138 (30.5)	1.72 ** (1.19–2.49)	0.55	1.80 ** (1.94–2.71)	0.60
	No	93 (57.1)	70 (42.9)	1.00		1.00	
Indebtedness	Yes	205 (65.9)	106 (34.1)	0.97 (0.70–1.36)	0.51	0.92 (0.63–1.35)	0.56
	No	203 (66.6)	102 (33.4)	1.00		1.00	

Notes: *n*—number of participants; %—percent of participants; OR—crude odds ratio; aOR—adjusted odds ratio for gender, age, education; CI—confidence interval for OR; AUC—area under the ROC curve. * *p* ≤ 0.05; ** *p* ≤ 0.01; *** *p* ≤ 0.001.

**Table 4:** Perceived health condition and socioeconomic status of private entrepreneurs from Wrocław (*N* = 616).

Variable		Perceived Health Condition		OR (±95% CI)	AUC	aOR (±95% CI)	AUC
		Average and Above *n* (%)	Below Average *n* (%)				
Marital status	Unmarried	82 (76.6)	25 (23.4)	1.41 (0.87–2.29)	0.52	0.90 (0.54–1.51)	0.65
	Married	356 (69.9)	153 (30.1)	1.00		1.00	
Persons per household	≤2 persons	148 (70.5)	62 (29.5)	0.57 (0.27–1.20)	0.54	0.92 (0.39–2.16)	0.79
	3–4 persons	248 (70.1)	106 (29.9)	0.56 (0.27–1.15)	0.53	0.61 (0.29–1.30)	0.62
	≥5 persons	42 (80.8)	10 (19.2)	1.00		1.00	
Per capita income	≥260 USD	424 (80.2)	105 (19.8)	21.06 *** (11.44–38.77)	0.69	23.84 *** (9.02–62.98)	0.76
	<260 USD	14 (16.1)	73 (83.9)	1.00		1.00	
Savings	Yes	339 (74.8)	114 (25.2)	1.92 ** (1.32–2.81)	0.57	2.57 *** (1.61–4.12)	0.63
	No	99 (60.7)	64 (39.3)	1.00		1.00	
Indebtedness	Yes	199 (64.0)	112 (36.0)	0.49 *** (0.34–0.70)	0.59	0.68 (0.46–1.01)	0.65
	No	239 (78.4)	66 (21.6)	1.00		1.00	

Notes: *n*—number of participants; %—percent of participants; OR—crude odds ratio; aOR—adjusted odds ratio for gender, age, education; CI—confidence interval for OR; AUC—area under the ROC curve. * *p* ≤ 0.05; ** *p* ≤ 0.01; *** *p* ≤ 0.001.

**Table 5:** Health-related quality of life in the physical and psychological domains and socioeconomic status of private entrepreneurs from Wrocław (*N* = 616).

Variable		Physical Domain		OR (±95% CI)	AUC	aOR (±95% CI)	AUC
		Average and Above *n* (%)	Below Average *n* (%)				
Marital status	Unmarried	81 (75.7)	26 (24.3)	0.89 (0.55–1.45)	0.51	0.55 * (0.31–0.97)	0.65
	Married	396 (77.8)	113 (22.2)	1.00		1.00	
Persons per household	≤2 persons	168 (80.0)	42 (20.0)	2.93 ** (1.54–5.60)	0.60	4.51 ** (2.09–9.71)	0.81
	3–4 persons	279 (78.8)	75 (21.2)	2.73 ** (1.49–50)	0.56	7.08 *** (3.27–15.31)	0.79
	≥5 persons	30 (57.7)	22 (42.3)	1.00		1.00	
Per capita income	≥260 USD	424 (80.2)	105 (19.8)	2.59 *** (1.6–4.19)	0.57	0.84 (0.32–2.18)	0.66
	<260 USD	53 (60.9)	34 (39.1)	1.00		1.00	
Savings	Yes	373 (82.3)	80 (17.7)	2.65 *** (1.77–3.95)	0.60	2.29 ** (1.37–3.84)	0.68
	No	104 (63.8)	59 (36.2)	1.00		1.00	
Indebtedness	Yes	206 (66.2)	105 (33.8)	0.25 *** (0.16–0.38)	0.66	0.34 *** (0.21–0.55)	0.71
	No	271 (88.9)	34 (11.1)	1.00		1.00	
**Variable**		**Psychological Domain**		**OR CI ± 95%**	**AUC**	**aOR CI ± 95%**	**AUC**
		**Average and Above *n* (%)**	**Below Average *n* (%)**				
Marital status	Unmarried	62 (57.9)	45 (42.1)	1.00 (0.66–1.52)	0.50	1.08 (0.68–1.71)	0.60
	Married	295 (58.0)	214 (42.0)	1.00		1.00	
Persons per household	≤2 persons	139 (66.2)	71 (33.8)	23.49 *** (8.14–67.76)	0.69	32.44 *** (10.61–99.16)	0.84
	3–4 persons	214 (60.5)	140 (39.5)	18.34 *** (6.47–52.00)	0.62	21.51 *** (7.39–62.58)	0.75
	≥5 persons	4 (7.7)	48 (92.3)	1.00		1.00	
Per capita income	≥260 USD	335 (63.3)	194 (36.7)	5.10 *** (3.05–8.54)	0.60	2.04 (1.00–4.17)	0.72
	<260 USD	22 (25.3)	65 (74.7)	1.00		1.00	
Savings	Yes	273 (60.3)	180 (39.7)	1.43 (0.99–2.04)	0.54	1.48 (0.95–2.30)	0.61
	No	84 (51.5)	79 (48.5)	1.00		1.00	
Indebtedness	Yes	183 (58.8)	128 (41.2)	1.08 (0.78–1.48)	0.51	1.11 (0.77–1.61)	0.60
	No	174 (57.0)	131 (43.0)	1.00		1.00	

Notes: *n*—number of participants; %—percent of participants; OR—crude odds ratio; aOR—adjusted odds ratio for gender, age, education; CI—confidence interval for OR; AUC—area under the ROC curve. * *p* ≤ 0.05; ** *p* ≤ 0.01; *** *p* ≤ 0.001.

**Table 6:** Health-related quality of life in the social and environmental domains and socioeconomic status of private entrepreneurs from Wrocław (*N* = 616).

Variable		Social Domain		OR (±95% CI)	AUC	aOR (±95% CI)	AUC
		Average and Above *n* (%)	Below Average *n* (%)				
Marital status	Unmarried	76 (71.0)	31 (29.0)	1.40 (0.89–2.21)	0.52	1.09 (0.69–1.74)	0.63
	Married	324 (63.7)	185 (36.3)	1.00		1.00	
Persons per household	≤2 persons	118 (56.2)	92 (43.8)	0.34 ** (0.17–0.71)	0.58	0.44 * (0.20–0.97)	0.76
	3–4 persons	241 (68.1)	113 (31.9)	0.57 (0.28–1.15)	0.53	0.46 * (0.21–0.98)	0.65
	≥5 persons	41 (78.8)	11 (21.2)	1.00		1.00	
Per capita income	≥260 USD	376 (71.1)	153 (28.9)	6.45 *** (3.89–10.70)	0.62	2.77 ** (1.38–5.57)	0.71
	<260 USD	24 (27.6)	63 (72.4)	1.00		1.00	
Savings	Yes	325 (71.7)	128 (28.3)	2.98 *** (2.06–4.31)	0.61	4.81 *** (2.94–7.88)	0.70
	No	75 (46.0)	88 (54.0)	1.00		1.00	
Indebtedness	Yes	187 (60.1)	124 (39.9)	0.65 * (0.47–0.91)	0.55	0.86 (0.58–1.26)	0.65
	No	213 (69.8)	92 (30.2)	1.00		1.00	
**Variable**		**Environmental Domain**		**OR (±95% CI)**	**AUC**	**aOR (±95% CI)**	**AUC**
		**Average and Above *n* (%)**	**Below Average *n* (%)**				
Marital status	Unmarried	83 (77.6)	24 (22.4)	0.89 (0.54–1.47)	0.51	0.66 (0.38–1.16)	0.64
	Married	405 (79.6)	104 (20.4)	1.00		1.00	
Persons per household	≤2 persons	165 (78.6)	45 (21.4)	0.77 (0.35–1.69)	0.52	0.95 (0.39–2.35)	0.80
	3–4 persons	280 (79.1)	74 (20.9)	0.79 (0.37–1.70)	0.51	0.78 (0.34–1.77)	0.65
	≥5 persons	43 (82.7)	9 (17.3)	1.00		1.00	
Per capita income	≥260 USD	463 (87.5)	66 (12.5)	17.40 *** (10.23–29.59)	0.72	16.83 *** (7.43–38.13)	0.81
	<260 USD	25 (28.7)	62 (71.3)	1.00		1.00	
Savings	Yes	404 (89.2)	49 (10.8)	7.75 *** (5.06–11.88)	0.72	6.27 *** (3.78–10.40)	0.79
	No	84 (51.5)	79 (48.5)	1.00		1.00	
Indebtedness	Yes	231 (74.3)	80 (25.7)	0.54 ** (0.36–0.80)	0.58	0.74 (0.48–1.14)	0.68
	No	257 (84.3)	48 (15.7)	1.00		1.00	

Notes: *n*—number of participants; %—percent of participants; OR—crude odds ratio; aOR—adjusted odds ratio for gender, age, education; CI—confidence interval for OR; AUC—area under the ROC curve. * *p* ≤ 0.05; ** *p* ≤ 0.01; *** *p* ≤ 0.001.

## Data Availability

Data available from the authors.
